# Integrated chromatin accessibility and DNA methylation analysis to reveal the critical epigenetic modification and regulatory mechanism in gonadal differentiation of the sequentially hermaphroditic fish, *Monopterus albus*

**DOI:** 10.1186/s13293-022-00484-6

**Published:** 2022-12-20

**Authors:** Qiaomu Hu, Zitong Lian, Xueping Xia, Haifeng Tian, Zhong Li

**Affiliations:** Yangtze River Fisheries Research Institute, Chinese Academy of Fishery Sciences, Wudayuan First Road 8, Wuhan, 430223 China

**Keywords:** Sex reversal, *Monopterus albus*, DNA methylation, Chromatin accessibility, Epigenetic modification

## Abstract

**Background:**

*Monopterus albus* is a hermaphroditic and economically farmed fish that undergoes sex reversal from ovary to testis via ovotestis during gonadal development. The epigenetic changes that are associated with gonadal development in this species remain unclear.

**Methods:**

We produced DNA methylome, transcriptome, and chromatin accessibility maps of the key stages of gonad development: ovary, ovotestis, and testis. The expression of the key candidate genes was detected using qRT-PCR and in situ hybridization and the methylation levels were analysed using bisulphite sequencing PCR. Promoter activity and regulation were assessed using dual-luciferase reporter assays.

**Results:**

Gonadal development exhibits highly dynamic transcriptomic, DNA methylation, and chromatin accessibility changes. We found that DNA methylation status, especially of the transcription start site, was significantly negatively correlated with gene expression while chromatin accessibility exhibited no correlation with gene expression during gonadal development. The epigenetic signatures revealed many novel regulatory elements and genes involved in sex reversal, which were validated. DNA methylation detection and site mutation of plastin-2 promoter, as a candidate gene, revealed that DNA methylation could impact the binding of transcription factor dmrt1 and foxl2 through methylation and demethylation to regulate plastin-2 expression during gonadal development.

**Conclusions:**

These data provide novel insights into epigenetic modification and help elucidate the potential molecular mechanism by which dynamic modification of DNA methylation plays a crucial role in gonadal development.

**Supplementary Information:**

The online version contains supplementary material available at 10.1186/s13293-022-00484-6.

## Highlights

- DNA methylation modification plays a more crucial role than chromatin accessibility in gonad development.
- DNA methylation in the transcription start site plays a key role in gonad development.
- DNA methylation of the plastin-2 promoter inhibited gene expression.
- Plastin-2 is positively regulated by *dmrt1* while negatively regulated by *foxl2.*

**Supplementary Information:**

The online version contains supplementary material available at 10.1186/s13293-022-00484-6.

## Introduction

Sex determination and sex differentiation are important developmental events in the life cycle of all sexually reproducing animals [1]. In fish, sex determination is complicated, and various types, including gonochorism and hermaphrodites, have been reported [2]. *Monopterus albus* is a freshwater fish with a snake-like body shape that is widely distributed in Asia from India to Asiatic Russia [3]. Owing to its high nutritional and medical value, this species is widely cultured in China, with up to 0.39 million tons being produced in 2019 [4]. *M. albus* is a hermaphroditic fish whose sex changes from female through intersex to male during its life cycle [5]. Recently, many studies from various perspectives were conducted to analyse the mechanism of sex reversal and abundant data were generated that support their insight [6–9]. However, the underlying mechanism of sex reversal remains unclear.

Epigenetic regulation plays a crucial role in sex determination and sex differentiation [10, 11]. DNA methylation, an important epigenetic modification, is generally associated with gene silencing [12] and play important roles in developmental process, especially sex differentiation [13]. In mice, DNA methylation levels of the promoter region of *sry* exhibited an inverse relationship with gene expression. DNA methylation levels were low in the E11.5 gonad while the expression level reached the peak [14]. In the red-eared slider turtle (*Trachemys scripta elegans*), KDM6B exhibits temperature-dependent sexually dimorphic expression, and knockdown of *Kdm6b* at 26 °C promoted male-to-female sex reversal [15]. In some fish, such as sea bass, temperature influences sex determination. In temperature-masculinized fish, high temperatures increased the *cyp19a* promoter methylation levels in females and methylation of *cyp19a* promoter decreased *cyp19a* expression [16]. Assay for Transposase-Accessible Chromatin using sequencing (ATAC-seq) is a novel technique used for the identification of open chromatin [17]. Chromatin is in an uncondensed state when open, and is accessible to gene transcriptional machinery and regulatory element binding. However, when condensed, chromatin is inactive and in a transcriptionally inaccessible state [18].

Here, we produced and analysed comprehensive DNA methylome, transcriptome, and chromatin accessibility maps of *M. albus* gonads over the course of gonad development. We found that chromatin accessibility was weakly correlated with the gene expression. On the contrary, a strongly negative correlation was observed between DNA methylation status and gene expression. Based on the expression profile, differentially expressed genes (DEGs) were divided into several groups, and we identified that DNA methylation of regulatory elements, especially the transcription start site (TSS), was strongly correlated with gene expression, which was up-regulated from ovary to ovotestis. Chromatin accessibility maps showed that these gene promoter regions were in low accessibility states which suggested that DNA methylation could play crucial roles in gonadal development. Plastin-2 was selected as the key candidate gene as per integration of low accessibility states, hypomethylation, and up-regulated expression. We determined that promoter methylation of plastin-2 was negatively correlated with gene expression in developing gonads and degenerated gonads treated with 17α-methyltestosterone (MT). We used zebularine to inhibit methyltransferase and found that the methylation status of three CpG sites could regulate plastin-2 gene expression. Finally, we found that transcription factor dmrt1 and foxl2 bind to the promoter of plastin-2 and were responsible for transcriptional activation of the plastin-2 gene. These data suggested that DNA methylation could impact the binding of transcription factor dmrt1 and foxl2 through methylation and demethylation to regulate plastin-2 expression during gonadal development.

## Methods

### Animals and samples

Rice field eel (*M. albus*) were obtained from the Breeding Center of Yangtze River Fisheries Research Institute, Chinese Academy of Fishery Sciences, China. Nine eels of three different growth stages (female, intersex, and male) were killed by MS222 administration according to Yangtze River Fisheries Research Institute Care Committee (No. 2013001) [6]. The gonads were collected and divided into three parts. One was preserved in 4% paraformaldehyde (pH 7.5) to prepare tissue sections; the second was frozen in liquid nitrogen and then stored at − 80 °C for RNA extraction; and the third was stored in ethanol for DNA extraction. The DNA and RNA was extracted as per the method described in a previous study [19]. Concentration of DNA and RNA were detected using an Agilent 2100 Bioanalyzer (Agilent Technologies) and the integrity was identified through agarose gel electrophoresis.

### Nanopore sequencing

Genomic DNA of the gonads from male, female, and intersex eels were extracted using the HiPure Tissue & Blood DNA Kit (Magen). The concentration and quality of DNA were detected using an Agilent 2100 Bioanalyzer (Agilent Technologies) and the integrity was detected using 1% agarose gel electrophoresis. Two microgrammes of gDNA was repaired using a NEB Next FFPE DNA Repair Mix kit (New England Biolabs). The ONT template prep kit (Oxford Nanopore Technologies) was used to prepare the template according the manufacturer’s instructions. The library was sequenced using the ONT sequencing reagent kit (Oxford Nanopore Technologies) according to the manufacturer’s instructions on the ONT PromethION48 platform with PromethION Flow Cells [20]. The fast5 format data were converted to fastq format for QC analysis using Basecall with ONT’s Guppy software [21]. The fastq data were further filtered to remove the adapters, short reads (length < 500 bp), and low-quality reads (MeanQual < 6).

### Identification and analysis of differentially methylated regions

Minimap2 [22] was used to analyse the sequence depth and alignment efficiency and to align the clean-reads to the reference genome. Nanopolish software with the Hidden Markov Model was used to detected the DNA methylation sites [23]. SMART2 software [24] was used to detect the differentially methylated region (DMR) and differentially methylated loci (DML). When the *P* value was < 0.05 and the methylation specificity was > 0.2, the CpG site or region was regarded as a DML or DMR. ChIPseeker software was used to locate the DML or DMR in the corresponding region in the genome [25].

### RNA-seq library generation and sequencing

Total RNA was extracted applying the TRIzol method according to the manufacturer’s instructions. NanoDrop 2000 (Thermo Fisher Scientific) and Bioanalyzer 2100 (Agilent Technologies) were used to determine the concentration and quality. RNA integrity was determined using 1% agarose gel electrophoresis. RNA-seq libraries were constructed in different gonad developmental stages (female, intersex, and male) in *M. albus.* The Ribo-Zero™ MagneticKit (Epicentre) was used to remove the rRNA, followed by reverse transcription of these RNAs to construct the cDNA library using NEBNext Ultra Directional RNALibrary Prep Kit (New England Biolabs). We then prepared paired-end sequencing on an Illumina Nova Seq6000 (Illumina San Diego). Clean data were obtained by removing reads containing adapters, reads containing poly-N, and low-quality reads from raw data. Q20, Q30, GC-content, and sequence duplication level of the clean data were calculated.

### Identification and analysis of differentially expressed genes

Differential expression analysis of two groups was performed using the DESeq R package (1.10.1) [26]. The threshold value was determined in multiple tests using the Benjamini–Yekutieli method to control the false discovery rate (FDR) [27, 28]. Criteria for DEGs were a false discovery rate < 10^−3^ and an expression level differing at least twofold between two groups.

### ATAC-seq library construction, sequencing, and mapping

For ATAC-seq library construction, gonads from three females, three intersex, and three males were collected. Approximately 50,000 cells were used for ATAC-seq library construction according to a previous description [29, 30]. The cells were washed with cold PBS and then suspended with cold lysis buffer (10 mM Tris, pH 7.4, 10 mM NaCl, 3 mM MgCl2, and 0.1% IGEPAL CA-630). The DNA was purified after incubation at 37 °C for 30 min with the Tn5 transposase. Transposed DNA fragments were used as the template for PCR amplification. The final DNA libraries were run on the Illumina NextSeq 500. Low-quality reads and adapters were removed and the Bowtie2 software was used to map the high-quality reads to the genome [31]. DeepTools v3.2 was used to map the density distribution of reads in the 3 kb intervals up- and down-stream of the TSS of each gene [32].

### Identification of ATAC peaks and DARs

Reads mapped to mitochondrial DNA and unplaced scaffolds were removed. MACS2 V2.1.1 [33] was used to identify ATAC peaks according to the following steps: removing redundant reads; then adjusting the read position; calculating peak enrichment; and estimating the empirical FDR. When the FDR was < 0.05, it was selected as the identified peak. To identify the differentially accessible regions (DARs), DiffBind version 2.6.6 [34] was used to detect the difference peak. The affinity score was calculated based on the read count which was then used as the input of the DESeq2 software [26] to screen for DARs in each group. When the fold change was > 1.5 and the *P* value was < 0.05, it was identified as a DAR.

### 17α-Methyltestosterone treatment

Two hundred and forty *M. albus* larvae at 60 days post-fertilization 60 per group, were used to produce sex reversal *M. albus* by sex steroid exposure as described in a previous study [35]. The control group underwent no treatment. Dry powders of MT were dissolved in 95% ethanol and then diluted in water. Larvae were immersed daily for 10–12 h in water containing MT at a concentration of 100 µg/L (MT1), 200 µg/L (MT2), and 300 µg/L (MT3). Forty mg of dry powders of MT were dissolved in 95% ethanol and then mixed with 200 g daphnia to a concentration of 200 µg/g MT. The mixture was kept at room temperature for 24 h and then stored at 4 °C. The trial continued for 2 months. The gonads were then collected and divided into three parts. One was preserved in 4% paraformaldehyde (pH 7.5) to prepare tissue sections, the second was frozen in liquid nitrogen and then stored at − 80 °C for RNA extraction, and the third was stored in ethanol for DNA extraction.

### Treatment of primordial gonadal cells with zebularine

Gonad tissues were collected from healthy *M. albus* specimens, and the tissues were washed with cold foetal bovine serum (FBS), the gonads were then cut into small pieces and placed on a 70-μm nylon mesh and gently pushed through the mesh with constant dripping of cold Dulbecco's modified Eagle medium (DMEM) (Thermo Fisher Scientific) containing 10% FBS (BioInd), 1% heparin and penicillin–streptomycin (P/S) (Thermo Fisher Scientific). The cell suspension was carefully collected and centrifuged at 500×*g* for 10 min at 4 °C. The supernatant was discarded and the cells were resuspended in 3 mL DMEM containing 10% FBS and 1% P/S. Finally, the cells were seeded in a 6-well plate (Corning) at a density of 1 × 10^6^ cells/well. The cells were incubated in a 5% CO2 incubator at 28 °C. After overnight culture, non-adherent cells were carefully washed off and the adherent cells were exposed to zebularine at concentrations of 50, 100, and 200 mg for 48 h. The cells were harvested for RNA preparation and DNA extraction to detect the change in expression profile and DNA methylation level.

### Plasmid constructs

Expression plasmids, pcDNA3.1-foxl2 and pcDNA3.1-dmrt1, were constructed from the pcDNA 3.1(+) plasmid using the double restriction endonucleases NheI and EcoRI (NEB). The promoter sequence of plastin-2 (XP_020477222) was obtained from the genomic database. Three deletion fragments (1575, 1074, and 436 bp) of the promoter were amplified from genomic DNA according to the designed primers (Additional file 1: Table S5), and cloned into the pGL3-basic vector (Promega) using KpnI and XhoI. Site-directed mutagenesis for the dmrt1 and foxl2 binding sites were performed using a Fast Site-Directed Mutagenesis Kit (TIANGEN) and the primers described in Additional file 1: Table S5.

### Cell culture, transfection, and dual-luciferase reporter assays

HEK293T cells were obtained from the Center of Animal Science and Animal Medicine, Shandong Agricultural University and cultured at 37 °C in DMEM (Thermo Fisher Scientific) containing 10% FBS (BioInd) and 1% P/S (Thermo Fisher Scientific) for 24 h before transfection. The cells were seeded onto the 24-well plates at a concentration of 1 × 10^5^ per well. The DMEM was removed and opti-MEM medium was added to incubate the cells. Five hundred nanogramme recombinant constructs and 50 ng pRL-TK were co-transfected into the cells in 400 µl opti-MEM medium using Lipofectamine™ 3000 (Invitrogen) according the manufacturer’s instructions and incubated at 37 °C for 6 h. Subsequently, the opti-MEM medium was removed and the cells was incubated for 48 h in DMEM. Cells were collected, and luciferase activity assays were performed using a Dual-Luciferase kit (Promega).

### In situ hybridization

To assess plastin-2 expression in gonadal cells, two pairs of primers were designed (Additional file 1: Table S5). A 433 bp cDNA fragment was amplified from the plastin-2 Open Reading Frame (ORF) domain. Primers were used to amplify the T7 promoter sequence synthetic probe, and PCR products were purified using a QIAquick Gel Extraction Kit (QIAGEN). Transcription was performed using the MEGAshortscript T7 High Yield Transcription Kit (Thermo Fisher Scientific) to obtain probes. In situ hybridization was performed as described in a previous study [36] using anti-digoxigenin-AP Fab fragments (Roche) as the antibody, while BCIP/NBT (Beyotime) was used to detect the positive signal.

### Quantitative real-time PCR

The expression of various tissues, developing gonads, and gonadal cells in *M. albus* were analysed by quantitative real-time PCR (qRT-PCR) according to a previous description [37]. *ef1a* was used as the internal control gene, cDNA was used as a template, tissues from three individuals were used, and each reaction was repeated three times. qRT-PCR was performed as follows: 95 °C for 30 s; 40 cycles of 95 °C for 5 s, 60 °C for 30 s, and 72 °C for 30 s; and then 72 °C for 5 min.

### Bisulphite PCR methylation analysis

Bisulphite PCR methylation analysis was conducted according to a previous description [38]. Genomic DNA from at least 15 individuals in one group was extracted using the TIANamp Genomic DNA Kit (Tiangen). Concentration of DNA was detected using an Agilent 2100 Bioanalyzer (Agilent Technologies) and the integrity was identified via agarose gel electrophoresis. Mixed DNA were treated using the DNA methylation kit (Zymo) following the manufacturer's protocol. Primers were designed using the online MethPrimer design software (http://www.urogene.org/methprimer/). PCR amplification was conducted using treated DNA as the template, and the purified DNA was cloned into the PMD-18T vector. Each group sequence had 15–20 positive clones. The methylation level was analysed using the DNA methylation analysis platform (http://services.ibc.uni-stuttgart.de/BDPC/BISMA/).

### Statistics

All statistical tests were performed using SPSS 22.0 (IBM). The difference in expression was analysed using one-way analysis of variance followed by Duncan multiple comparison tests. Significance was set at *P* < 0.05, and different letters indicated significantly difference. Differences in mean expression level and methylation level among groups were determined using an independent sample t-test. Differences in the ratio of methylated to unmethylated CpG at each site were assessed using a Chi-square test followed by Fisher's exact test. A P value of less than 0.05 was considered significant.

## Results

### Genome-wide DNA methylation level profiling

**Fig. 1:**
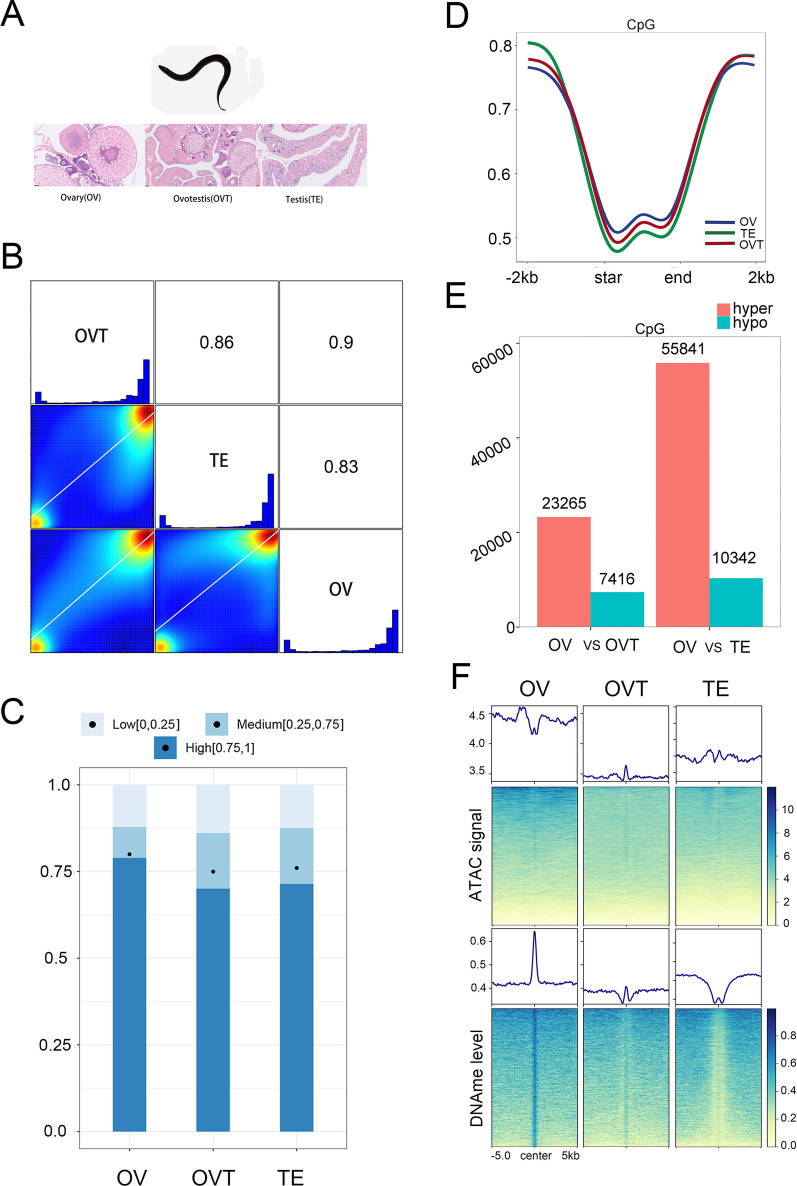
DNA methylation dynamic alteration during the gonadal development. **A** Scheme of HE section from developing gonad. **B** Pearson correlation coefficient of genome-wide CpG methylation among the developing gonad. **C** Global CpG methylation levels and total CpG with low (0,0.25), medium (0.25,0.75) and high (0.75,1) methylation level during gonadal development. **D** Distribution of genome-wide CpG methylation level of each stage of gonadal cell. **E** Number of DMRs identified between the two groups. **F** ATAC-seq signals and DNA methylation levels over 10-kb regions centred on a total of 15496 hypo-DMRs

To understand the DNA methylation changes during *M. albus* gonadal development, we collected the samples from the three important developmental stages female (ovary), intersex (ovotestis), and male (testis) (Fig. 1A). Whole-genome bisulphite sequencing was conducted and 36.1 GB, 31.7 GB, and 32.9 GB clean data were obtained from the ovary, ovotestis, and testis groups, respectively (Additional file 1: Table S1). The raw sequence data were deposited in the National Genomics Data Center [39] under the accession number CRA007182. The string average depths were 45.1 (ovary), 39.5 (ovotestis), and 41.1 (testis). All clean-reads were assembled and mapped to the *M. albus* reference genome [40]. Means of 99.65% (ovary), 99.57% (ovotestis), and 99.57% (testis) reads were mapped to the genome (Additional file 1: Table S1). To detect the DNA methylation dynamics, global CpG methylation levels during gonadal development were analysed and a scale of methylation level between 0.75 and 1 in ovaries was higher than that in ovotestes and testes. However, a scale of methylation level between 0.25 and 1 showed no dramatic differences among the ovary, ovotestis, and testis (Fig. 1C). The distribution of global CpG methylation levels in ovary, ovotestis, and testis showed a similar bimodal distribution with a peak outside gene region (Fig. 1D). To further detect the DNA methylation dynamics in gonadal development, 23,265 hyper- and 7416 hypo-DMRs in the OVT/OV groups, and 55,841 hyper- and 10,342 hypo-DMRs in the TE/OV groups (Fig. 1E). Taken together, 15,496 hypo-DMRs were obtained. To study whether hypo-DMRs were associated with regulation activity, an ATAC-seq library was constructed from the same samples containing three ovaries, three ovotestis, and three testes. DMR exhibited much lower ATAC-seq signals than their neighbouring region in the OV group, while ATAC-seq signals were absent in the OVT and TE groups (Fig. 1F). Dynamic change of the methylation profile from ovary to testis showed that methylation level decreased gradually (Fig. 1F). These results suggested that DNA methylation may play a key role in the development of ovary to testis.

### Genes activation associated with the TSS methylation status

**Fig. 2:**
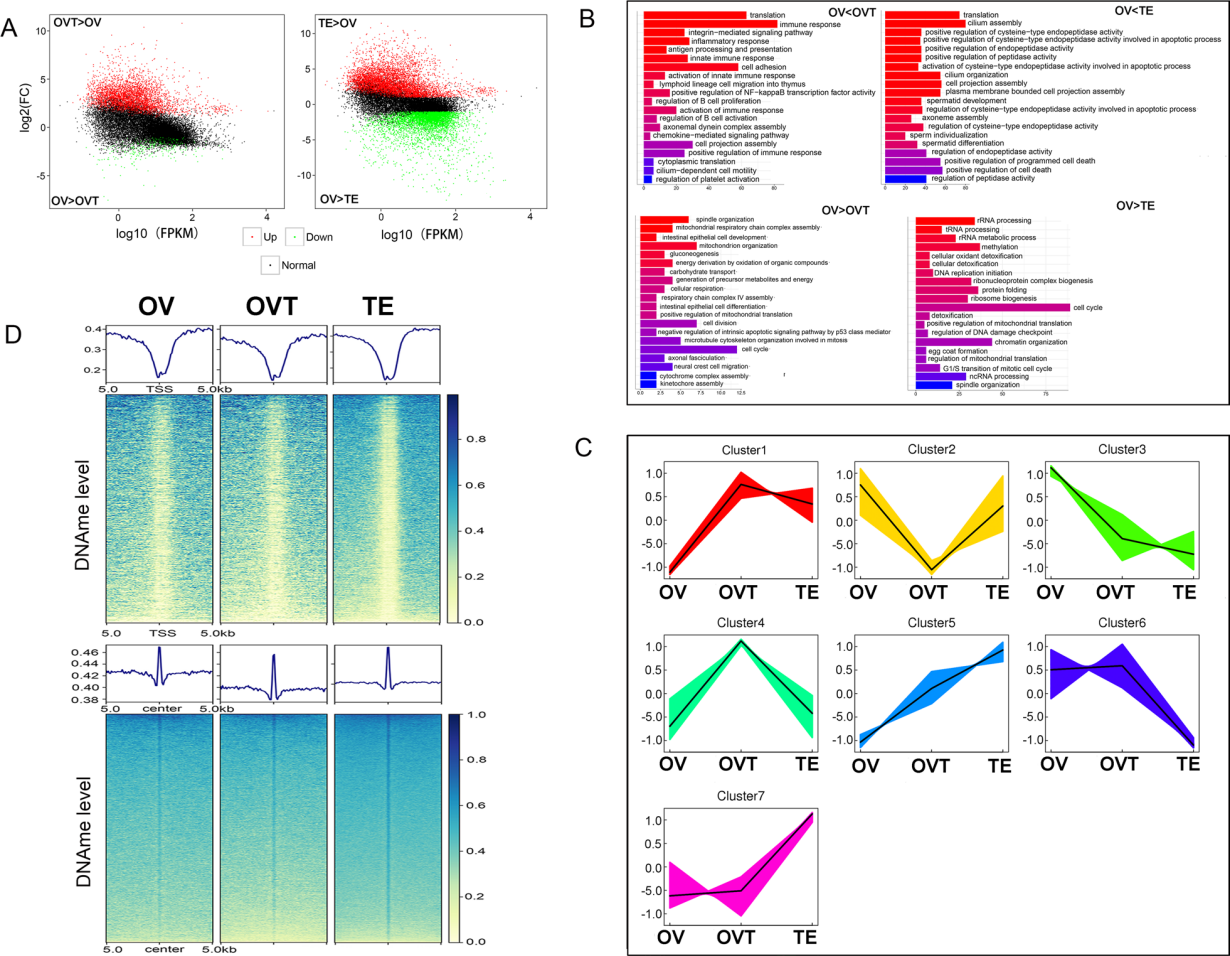
DNA methylation changes in TSS region activated gene expression. **A** MA plots for differentially expressed genes during gonadal development. Black dots represent genes with no significantly change. Red and green dots represent significantly up-regulated and down-regulated genes during gonadal development, respectively (*P* < 0.05). **B** Gene ontology terms associated with significantly differentially expressed genes. **C** Different expression profile during the gonadal development. **D** DNA methylation level over 10-kb region centre and promoter of the differentially expressed genes from cluster 4

To study the relationship between DNA methylation status and transcriptomic dynamics during gonadal development, RNA-seq was conducted using the same samples containing three ovaries, three ovotestis and three testes. A total of 4527 DEGs containing 469 down-regulated and 4058 up-regulated genes displayed > twofold differential transcript abundance in the OVT/OV group (Fig. 2A). In the TE/OV group, a total of 11,853 DEGs containing 5523 down-regulated and 6330 up-regulated genes (Fig. 2A). The up-regulated genes in the OVT/OV group were enriched in biological functions including translation, cell adhesion, spermatid differentiation, and immune response (Fig. 2B). All DEGs were divided into seven groups according to the expression profiles (Fig. 2C). Nine hundred and three genes in cluster 4 exhibited up-regulated expression from ovary to ovotestis which was then down-regulated from ovotestis to ovary. The expression profile of cluster 4 suggested that these genes could play key roles in sex reversal. To examine DNA methylation dynamics of regulatory elements associated with the expression of these genes, DNA methylation changes were analysed for these gene promoters and the centre region. DNA methylation levels showed no remarkable difference in promoter and centre regions among the groups. However, in the promoter region, the methylation status was hypo while in the centre region, the methylation status was hyper (Fig. 2D). These results suggested that the promoter region methylation status might regulate the expression of these genes.

### Gene activation is not associated with decreasing chromatin accessibility

**Fig. 3:**
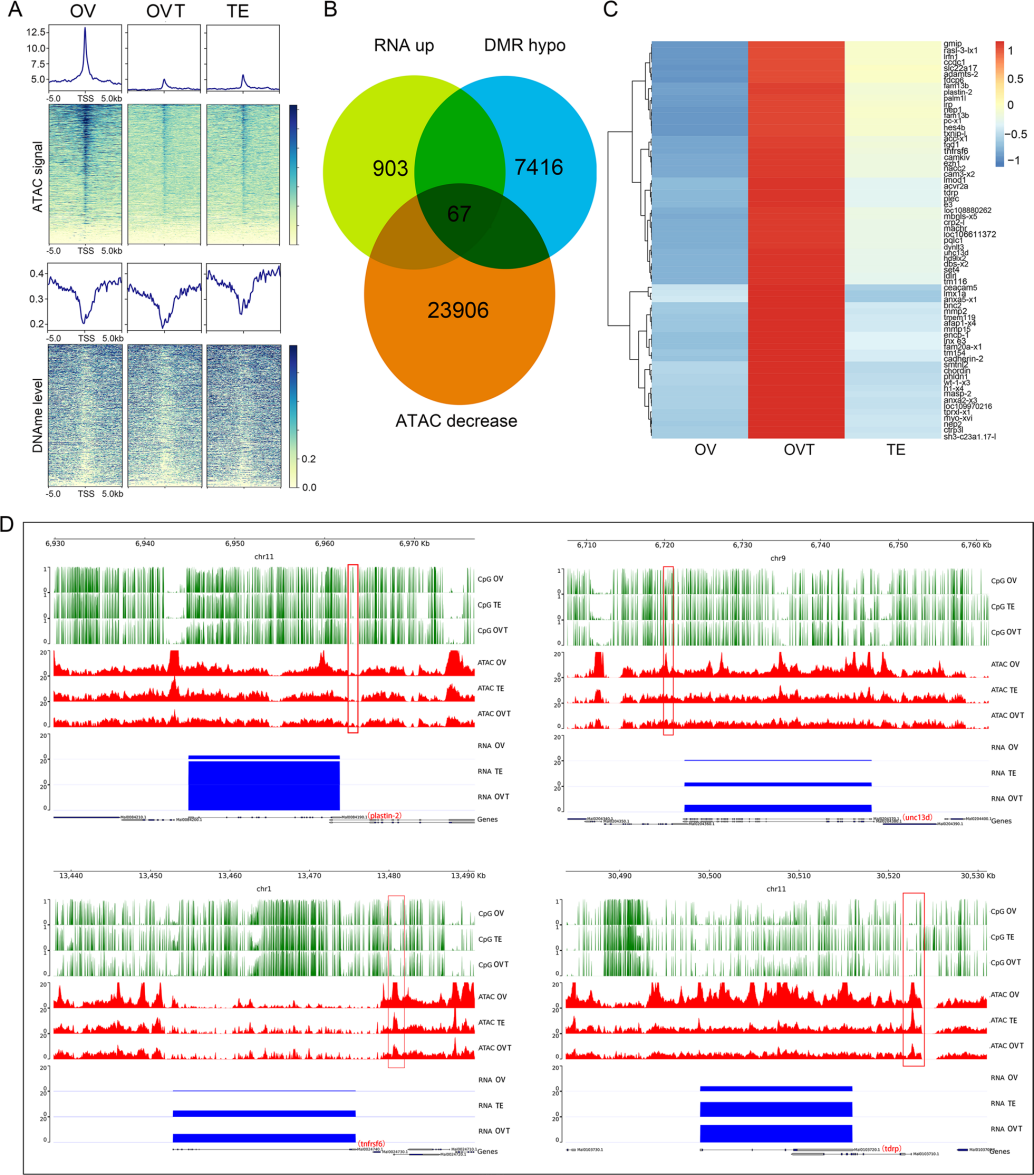
Gene activation with lose of the chromatin accessibility. **A** DNA methylation and ATAC signal changes over 10-kb promoter region of the differentially expressed genes from cluster 4. **B** Overlap genes of DMR hypo, ATAC decrease and RNA up-regulated from cluster 4. **C** Heatmap of the 67 overlap genes expression profile. **D** Identified candidates of sex-reversal related genes. Red boxes indicate DARs changes during sex reversal

To test the hypothesis that accessible chromatin is key in regulating gene transcription, ATAC-seq libraries from the same samples were generated. The raw sequence data were deposited in the National Genomics Data Center under the accession number CRA007367. About 100 MB of total clean data were obtained from each sample and > 95% of reads were mapped to the genome (Additional file 1: Table S2). The peak number in the three groups ranged from 153,317 to 306,358. We found that about 25% of ATAC peaks were in the promoter region (3 kb region around the TSS), 35% of ATAC peaks were distally intergenic to TSS (Additional file 1: Table S3) while no peak was found in the 3ʹ- and 5ʹ-UTRs. To study the dynamics of chromatin accessibility during gonadal development, the differential accessibility regions (DARs) were identified and a total of 53,540 and 41,010 DARs exhibited > twofold differential accessibility in the OVO/OV and TE/OV groups (Additional file 1: Table S4). To determine whether these dynamic chromatin accessibility changes were associated with gene expression in gonadal development, ATAC signal and DNA methylation level of 903 genes from cluster 4 were analysed and it was found that ATAC-seq signals were decreased from ovary to ovotestis and the expression profile was increased while the DNA methylation level was decreased (Fig. 3A). Sixty-seven overlapped genes were obtained from hypo-DMRs, ATAC-decreased, and the genes from cluster 4 (Fig. 3B). These gene expressions were up-regulated from ovary to ovotestis and down-regulated from ovotestis to testis (Fig. 3C). Eleven out of 67 candidate genes were verified and only seven genes (*slc22a17*, *pmp-22*, *tnfrs6*, plastin*-*2, *tdrp*, *pqlc1*, and *unc13d*) displayed the expression profile of cluster 4 (Additional file 1: Fig. S1). Integrative Genomics Viewer showed that the RNA transcription level was negative with the ATAC-seq peak and DNA methylation level for plastin-2 gene, *unc13d*, *tnfrs6*, and *tdrp* (Fig. 3D). These results support that chromatin accessibility was not responsible for activating gene expression while DNA methylation might play a key role in gonadal development.

### DNA methylation was associated with plastin-2 expression in developing gonads

**Fig. 4:**
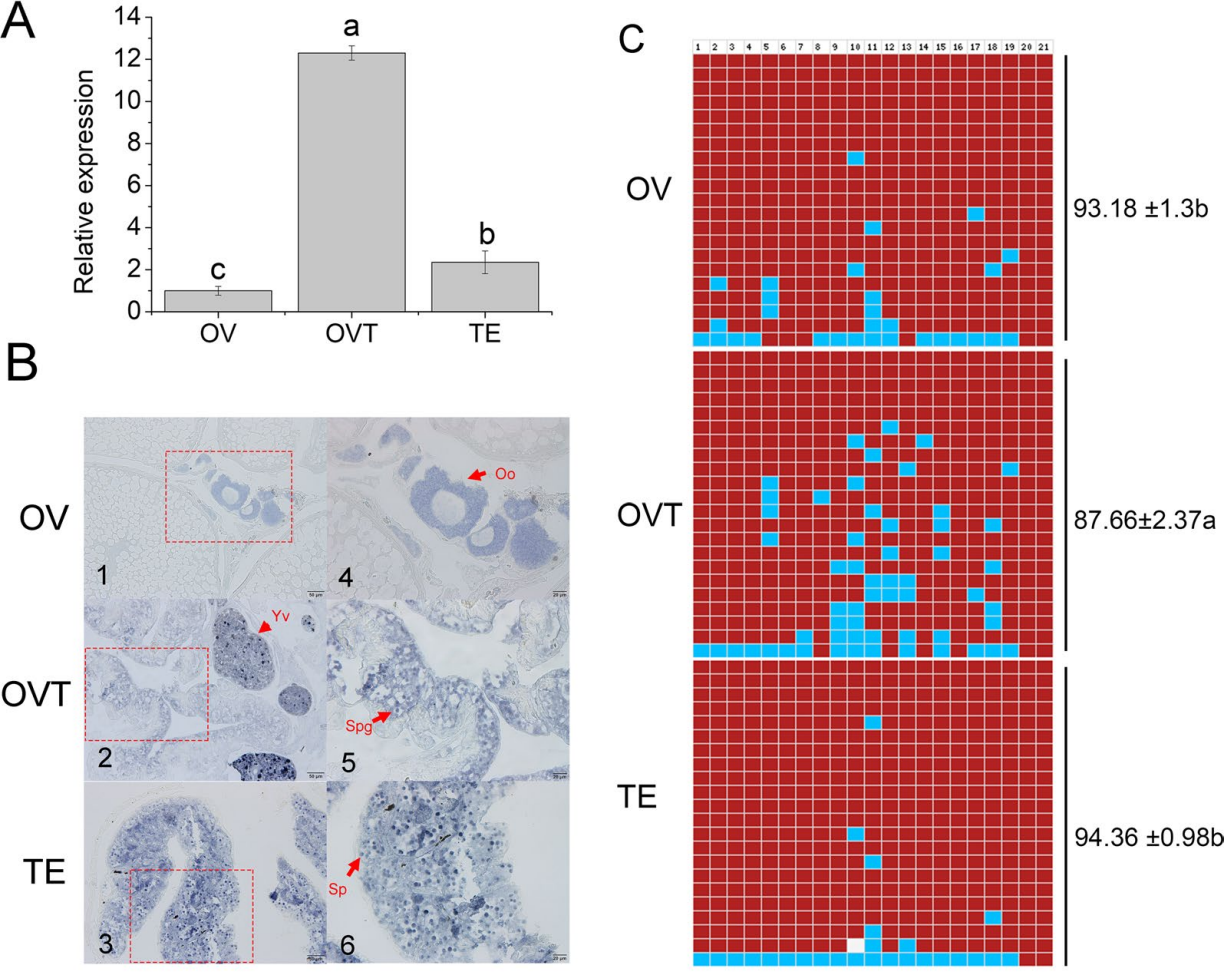
Expression and methylation level of plastin-2 in ovary, ovariotestis and testis of *Monopterus albus*. **A** Expression profile of plastin-2 in ovary, ovariotestis and testis detected by qRT-PCR; **B** RNA expression of plastin-2 in ovary, ovariotestis and testis detected by in situ hybridization; **C** DNA methylation level of plastin-2 in ovary, ovariotestis and testis of *Monopterus albus*. Oo: oocytes; Sp: spermatocytes; Yv: yolk vesicle; Spg: spermatogonia. 4,5,6 is the large magnification of frame area in 1,2,3, respectively

To detect the sex reversal mechanism of plastin-2 during gonadal development, we analysed the association between RNA transcription and DNA methylation. We firstly analysed the sequence of the promoter region (GenBank accession no: ON692924) using MethPrimer software and found a region around the TSS with high CpG content; the methylation primers were designed according this sequence (Additional file 1: Table S5). We found that plastin-2 expression was significantly higher in the ovotestis than in the ovary and testis (*P* < 0.05, Fig. 4A). To detect the distribution of plastin-2 in gonads, in situ hybridization was conducted to identify the expression of plastin-2 in ovary, testis, and ovotestis cells while the sense probe had no signal (Additional file 1: Fig S2). We found a strongly positive signal in granulosa cells and cytoplasm in ovaries; in ovotestis, plastin-2 was strongly expressed in granulosa cells and spermatogonia whereas, in testis, plastin-2 was strongly detected in spermatocytes and spermatids (Fig. 4B). The methylation status among the same samples during gonadal development were detected (Additional file 1: Table S6) and we found that methylation levels were significantly higher in the testis and ovary (94.36% and 93.18%, respectively), than in ovotestis (87.66%; *P* < 0.05, Fig. 4C). The results suggested that methylation status was negatively associated with the expression level.

### Expression and DNA methylation status of plastin-2 after MT treatment

**Fig. 5:**
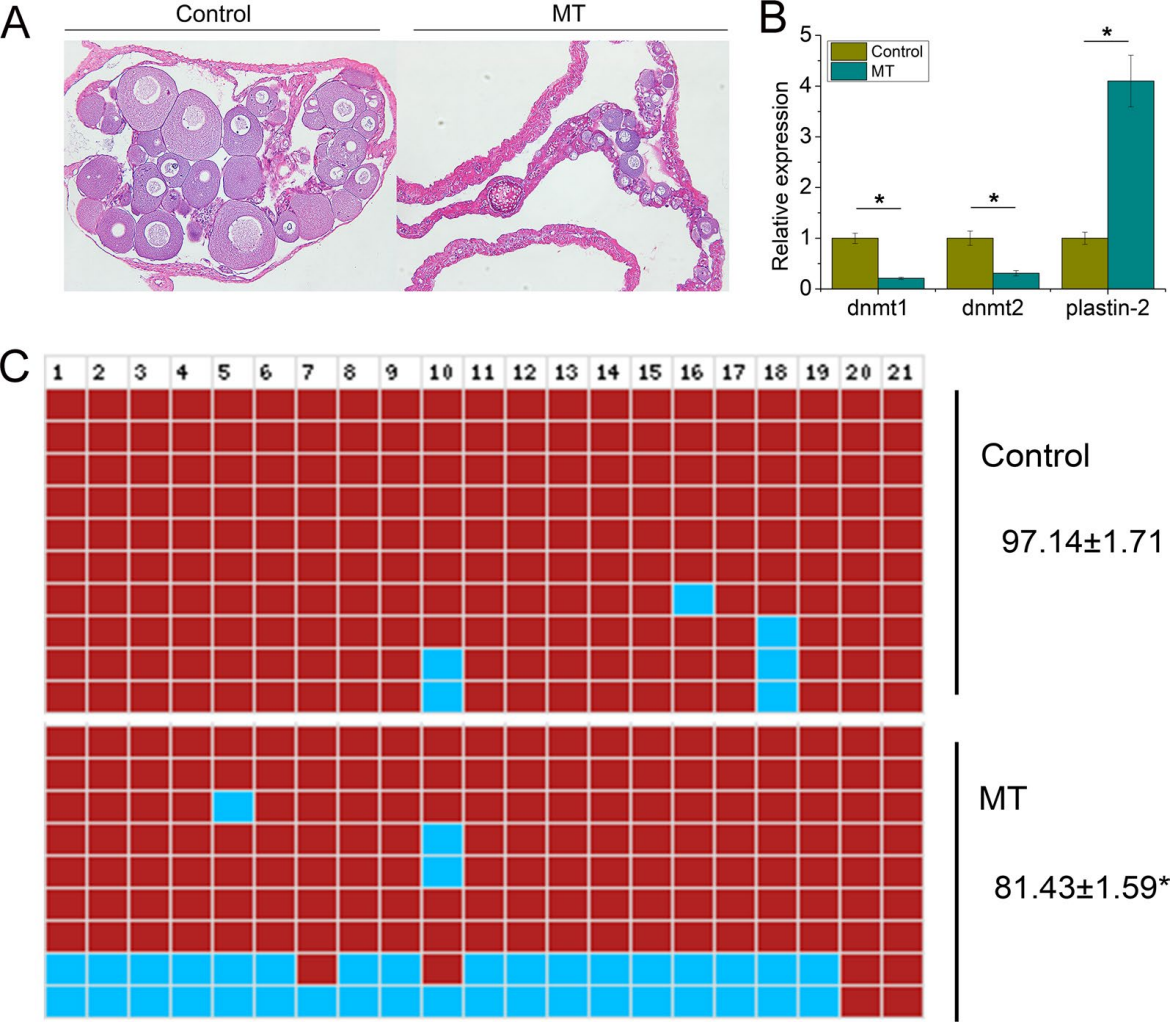
Expression and DNA methylation level of plastin-2 in ovary and degenerated ovary treated by MT. **A** Histology sections of ovary and degenerated ovary treated by MT; **B** expression profile of dnmt1, dnmt2 and plastin-2 between the ovary and degenerated ovary treated by MT; **C**. methylation level of plastin-2 promoter between the ovary and degenerated ovary treated by MT

To further examine the sex reversal mechanism of plastin-2, MT was used to treat the larvae for 2 months. Histological examination showed that the ovary was degenerated after MT treatment (Fig. 5A). The number of oocytes and cellular volume decreased. Additionally, connective tissues were increased (Fig. 5A). Expression of dnmt1 and dnmt2 were significantly decreased while plastin-2 expression was significantly increased (*P* < 0.05, Fig. 5B). The results suggest that DNA methylation status undergoes changes. Bisulphite sequencing PCR (BSP) examined the DNA methylation status after MT treatment (Additional file 1: Table S7). We found that the DNA methylation level was significantly decreased (*P* < 0.05, Fig. 5C).

### DNA methylation could regulate plastin-2 expression

**Fig. 6:**
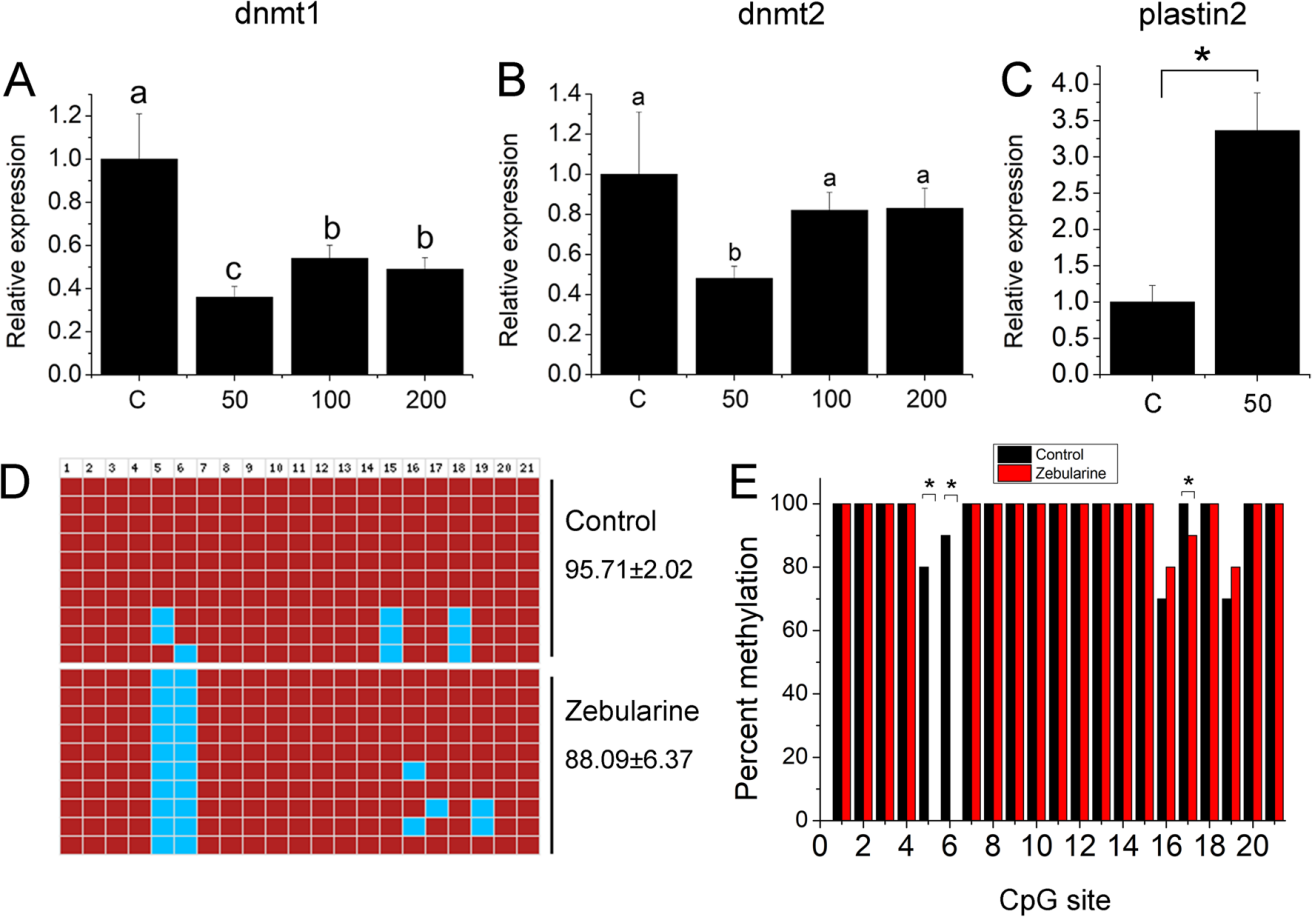
Expression and methylation level of plastin-2 in primordial gonadal cells treated by zebularine. **A** Expression profile of dnmt1 in various concentrations of zebularine; **B** expression profile of dnmt2 in various concentrations of zebularine; **C** expression profile of plastin-2 at concentrations of 50 mg zebularine; **D** methylation level of plastin-2 promoter after treated by zebularine; **E**. comparison of methylation level in each CpG sites between zebularine and control group

To detect the role of promoter methylation in plastin-2 expression, zebularine was used to inhibit the methylation level in the primordial gonadal cells. We found that dnmt1 and dnmt2 expression were significantly decreased at a concentration of 50 mg compared with that of the other genes (*P* < 0.05, Fig. 6A, B), while expression of plastin-2 was significantly increased (*P* < 0.05, Fig. 6C). DNA methylation level was lower than the control group after zebularine treatment but was not significantly different (*P* > 0.05, Fig. 6D), while methylation level of CpG sites 5,6, and 17 were significantly decreased in the zebularine group (*P* < 0.05, Additional file 1: Table S8, Fig. 6E). These results suggested that zebularine inhibited the methylation level of CpG sites to promote gene expression.

### Dmrt1 and foxl2 activate plastin-2 promoter

**Fig. 7:**
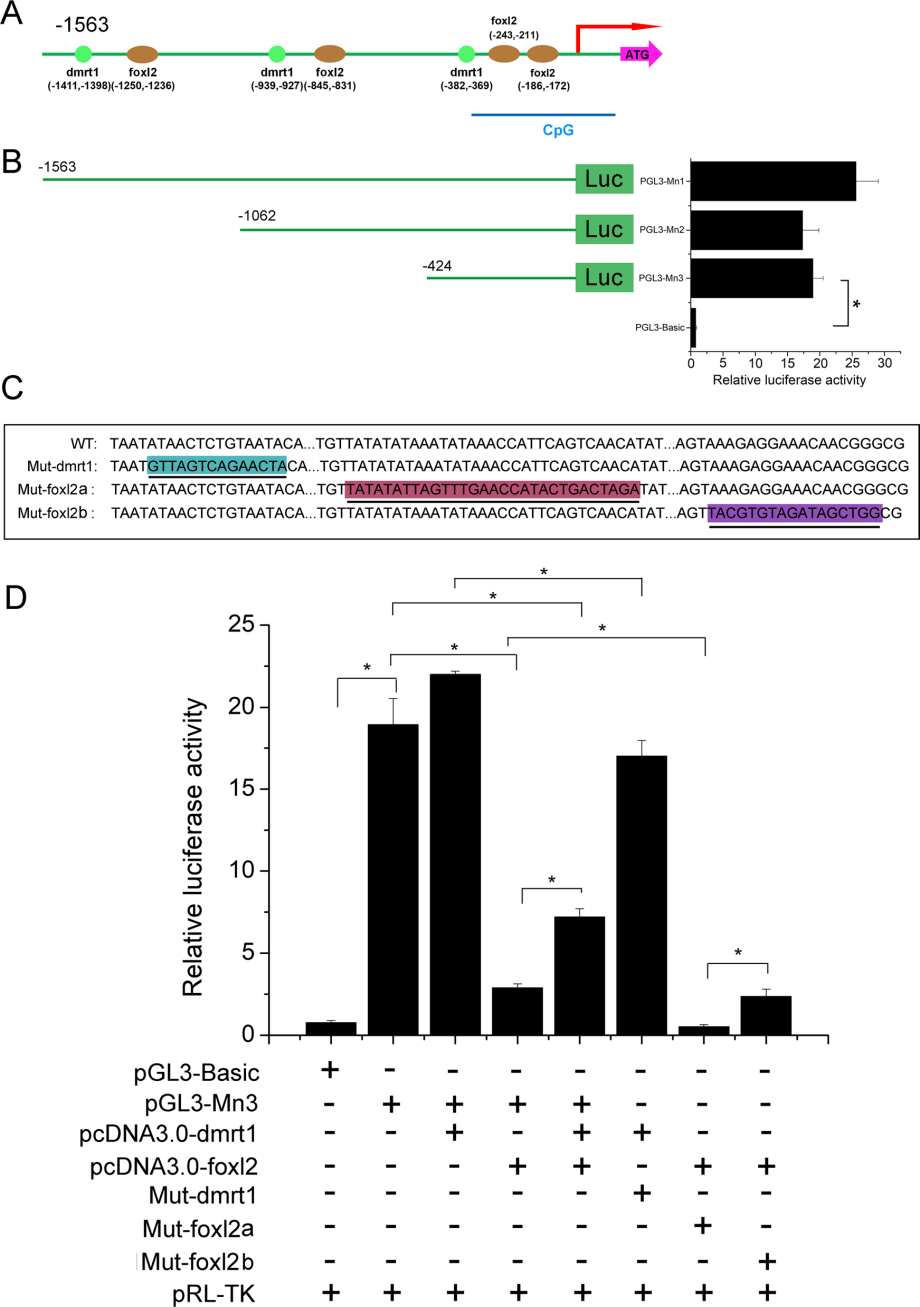
Luciferase assay detected the binding sites and activation of transcriptional factor. **A** Schematic showing the dmrt1 and foxl2 binding sites and CpG sites. **B** Luciferase assay showing the activity of deletions constructs. **C** Schematic showing the mutation of dmrt1 and foxl2, and the wild type. **D** Luciferase assay showing site mutation of promoter in 293T cells. The mean ± SEM was from three independent experiments, *P < 0.05 shows significantly difference

To explore the regulatory elements, potential binding sites for transcription factors were predicted using the JASPAR online software. Several sex-related transcription factor binding sites were found in the promoter region (Fig. 7A). To determine the exact binding sites, luciferase reporter assays with a series of deletions were conducted and the luciferase activities were significantly higher than the basic group in the three deletion constructs (Fig. 7B). Luciferase activities exhibited that key regulatory elements ranged from − 1 to − 424 which includes one dmrt1 and two foxl2 binding sites (Fig. 7B). To determine the potential role of the transcriptional factors dmrt1 and foxl2 in the plastin-2 promoter, site mutants were constructed using the PGL3-Mn3 plasmid as the template. Three mutants including Mut-dmrt1, Mut-foxl2a, and Mut-foxl2b were obtained (Fig. 7C). Luciferase activities were significantly decreased after the dmrt1 binding site mutation in group of pGL3-Mn3 + pcDNA3.0-dmrt1 (*P* < 0.05, Fig. 7D). Additionally, we found that the foxl2a binding site mutation caused significant down-regulation of the luciferase activity in the pGL3-Mn3 + pcDNA3.0-foxl2 group (*P* < 0.05), while the foxl2b binding site mutation caused no significant difference (*P* > 0.05, Fig. 7D). The findings suggested that dmrt1 and foxl2 could bind to activate the plastin-2 promoter, while mutation of any of them caused a decrease in luciferase activity, except for the foxl2b binding site.

## Discussion

Epigenetic modifications, including DNA methylation and chromatin accessibility, have been proposed as the molecular mechanisms underlying sex differentiation [15, 41, 42]. In the present study, we used *M. albus,* a good model for studying sex reversal [43], to investigate the potential role of DNA methylation and chromatin accessibility in gonadal development. Gonads from three key stages of the developmental process were selected to investigate with multiple epigenomics assays. We found a weak correlation between gene expression and chromatin accessibility during sex reversal. However, we found that DNA methylation status had a strong negative correlation with gene expression in the dynamic changes. The observation suggested that DNA methylation might play a crucial role during sex reversal. Another study also exhibited that DNA methylation and chromatin accessibility work together to regulate gene expression in zebrafish regeneration [44].

To detect how epigenetic modification affects regulatory elements to alter gene expression, we detected hundreds of genes activated in sex reversal using RNA-seq. Epigenetic modification of regulatory elements was identified and we found that chromatin accessibility was also weakly responsible for gene expression while DNA methylation status was strongly negatively correlated with gene expression, especially for the TSSs. These regulatory elements were in a hypomethylated but low accessibility state in sex reversal. Whether epigenetic modifications other than DNA methylation in these regulatory elements contribute to sex reversal should be further studied. Global epigenome profiling not only has advantages for detecting genomic regulatory elements but it also identified important regulatory genes. To further explore the association of DNA methylation with sex reversal, up-regulated genes with hypomethylated profiles of promoter and low accessibility states were selected as the putative candidate genes for further study.

Plastin-2, called L-plastin, an actin-binding protein [45] known for regulating T-cell adhesion and immune synapse formation [46]. Recently, another study showed that plastin-2 is expressed in the ovary and plays a crucial role in reproduction [47, 48]. Additionally, we found that the binding sequence of oestrogen receptor was in the promoter region of plastin-2 and its expression was up-regulated after dihydrotestosterone and oestradiol induction [49, 50]. However, the function of plastin-2 involved in sex differentiation was unclear. RNA-seq showed that plastin-2 expression was significantly up-regulated from ovary to ovotestis the gonadal development while the promoter was hypomethylated and in a low accessibility state. The results suggested that DNA methylation was strongly negatively associated with gene expression. To further detect whether DNA methylation was closely related to gene expression, we detected the DNA methylation level of plastin-2 promoter and plastin-2 gene expression during the key stages of gonadal development. We found that plastin-2 gene expression was negatively correlated with promoter methylation status, and that plastin-2 was expressed in granulosa cells and cytoplasm in ovaries whereas in spermatocytes and spermatids in testes. After larvae were treated with MT, we found that ovaries were degenerated and that plastin-2 expression was significantly up-regulated with a hypomethylated promoter profile. In another study in humans, plastin-2 expression was up-regulated after oestradiol and dihydrotestosterone treatment [49]. Additionally, dnmt1, dnmt2, and DNA methyltransferase1 and 2 expression was down-regulated, which suggested that methyltransferase could be involved in sex reversal. To detect whether methylase activity inhibits up-regulation of plastin-2 expression during sex reversal, zebularine, which inhibits DNA methyltransferase during replication through covalent binding [51], was used to treat primordial gonadal cells to inhibit DNA methylation. The methylation level was low in the treated group with no significant difference. We determined three CpG sites whose methylation levels were significantly down-regulated and were negatively associated with plastin-2 expression. To determine the exact binding sites that activate plastin-2 expression in the promoter, binding sites of transcription factors were detected and luciferase activity was analysed We found dmrt1 and foxl2 can bind to the plastin-2 promoter and were responsible for transcriptional activation of the plastin-2 gene. Dmrt1 and foxl2 are known as the marked genes in sex differentiation that have been reported in many species [52–54]. In addition, many studies have determined that promoter methylation can inhibit the binding of transcription factors in the gene promoter region, resulting in the decline of gene transcription level or even termination of transcription, to regulate gene expression [55, 56]. Taken together, these data suggest that DNA methylation could impact the binding of transcription factor dmrt1 and foxl2 through methylation and demethylation to regulate plastin-2 expression during gonadal development.

## Perspectives and significance

*Monopterus albus* is a hermaphroditic fish that undergoes sex reversal from ovary to testis via ovotestis during gonadal development which is consider an ideal model for genetic research. The present study on the gonadal development of *M. albus* provides novel insights from epigenetic modification to help elucidate the potential molecular mechanism. It will facilitate the sex control and biomarker exploration in *M. albus.* In the future, epigenetic markers will be explored to identify the un-reversal female to improve the fecundity which exhibited low level in the normal female due to the small body size. Function studies on epigenetic modification are needed to elucidate the molecular mechanism of *M. albus* gonadal development and to reveal the sex differentiation mechanism of the hermaphroditic animal.

## Supplementary Information

**Additional file 1: Fig. S1.** Verification of the candidate genes. **Fig. S2.** In situ hybridization using sense probe. **Table S1.** Summary of the clean reads of the DNA methylation. **Table S2.** Summary of the ATAC-seq. **Table S3.** Distribution of peak of ATAC. **Table S4.** Differentially peak of ATAC in different group. **Table S5.** Primers and sequences used in this study. Table S6. Methylation level of each site for the ovary (OV), Ovotestis (OVT) and testis (TE) groups. **Table S7.** Methylation level of each site for the control and MT groups. **Table S8.** Methylation level of each site for the control and zebularine (ZE) groups.

## Data Availability

All data relevant to the study are included in the article or uploaded as supplementary information. The raw sequence data reported in this paper have been deposited in the National Genomics Data Center (National Genomics Data Center and Partners, 2020), Beijing Institute of Genomics (China National Center for Bioinformation), Chinese Academy of Sciences, under the accession number CRA007367 for ATAC data and CRA007182 for DNA methylation data. All data needed to evaluate the conclusions in the paper are present in the paper and/or the Supplementary Materials.

## Abbreviations

DMR: Differentially methylated region
DML: Differentially methylated loci
ATAC-seq: Assay for Transposase-Accessible Chromatin
DEG: Differentially expressed gene
TSS: Transcription start site
OV: Ovary
OVT: Ovotestis
TE: Testis
MT: 17α-Methyltestosterone
DAR: Differential accessibility regions
BSP: Bisulfite sequencing PCR
FDR: False discovery rate
FBS: Foetal bovine serum

## References

[1] Koopman P (2001). Gonad development: signals for sex. Curr Biol.

[2] Devlin RH, Nagahama Y (2002). Sex determination and sex differentiation in fish: an overview of genetic, physiological, and environmental influences. Aquaculture.

[3] Supiwong W, Pinthong K, Seetapan K, Saenjundaeng P, Bertollo LAC, de Oliveira EA (2019). Karyotype diversity and evolutionary trends in the Asian swamp eel Monopterus albus (Synbranchiformes, Synbranchidae): a case of chromosomal speciation?. BMC Evol Biol.

[4] CSY, China Statistical Yearbook. China statistical. Beijing: Publishing House; 2019

[5] Liu C (1944). Rudimentary hermaphroditism in the symbranchoid eel. Monopterus Javanensis. Sinensia..

[6] Hu Q, Xiao Q, Tian H, Li D, Li Z (2021). Crucial role of dead end gene for primordial germ cell survival in rice field eel (*Monopterus albus*). Theriogenology.

[7] Chen H, Liu H, Li R, Lin X, Luo D (2021). Blood cell identification and hematological analysis during natural sex reversal in rice field eel (*Monopterus albus*). Aquaculture.

[8] Yan T, Lu H, Sun C, Peng Y, Meng F, Gan R (2021). Nr5a homologues in the ricefield eel *Monopterus albus*: alternative splicing, tissue-specific expression, and differential roles on the activation of cyp19a1a promoter in vitro. Gen Comp Endocrinol.

[9] Wang X, Lai F, Xiong J, Zhu W, Yuan B, Cheng H (2020). DNA methylation modification is associated with gonadal differentiation in *Monopterus albus*. Cell Biosci.

[10] Kuroki S, Tachibana M (2018). Epigenetic regulation of mammalian sex determination. Mol Cell Endocrinol.

[11] Stévant I, Nef S (2019). Genetic control of gonadal sex determination and development. Trends Genet.

[12] Kojima-Kita K, Kuramochi-Miyagawa S, Nagamori I, Ogonuki N, Ogura A, Hasuwa H (2016). MIWI2 as an effector of DNA methylation and gene silencing in embryonic male germ cells. Cell Rep.

[13] Smallwood SA, Kelsey G (2012). De novo DNA methylation: a germ cell perspective. Trends Genet.

[14] Nishino K, Hattori N, Tanaka S, Shiota K (2004). DNA methylation-mediated control of Sry gene expression in mouse gonadal development. J Biol Chem.

[15] Ge C, Ye J, Weber C, Sun W, Zhang H, Zhou Y (2018). The histone demethylase KDM6B regulates temperature-dependent sex determination in a turtle species. Science.

[16] Navarro-Martín L, Viñas J, Ribas L, Díaz N, Gutiérrez A, Di Croce L (2011). DNA methylation of the gonadal aromatase (cyp19a) promoter is involved in temperature-dependent sex ratio shifts in the European sea bass. PLoS Genet.

[17] Buenrostro JD, Wu B, Chang HY, Green leaf WJ (2015). ATAC-seq: a method for assaying chromatin accessibility genome-wide. Curr Protoc Mol Biol.

[18] Yan F, Powell DR, Curtis DJ, Wong NC (2020). From reads to insight: a hitchhiker’s guide to ATAC-seq data analysis. Genome Biol.

[19] Hu Q, Ao Q, Tan Y, Gan X, Luo Y, Zhu J (2020). Genome-wide DNA methylation and RNA analysis reveal potential mechanism of resistance to *Streptococcus agalactiae* in GIFT strain of Nile Tilapia (*Oreochromis niloticus*). J Immunol.

[20] Deamer D, Akeson M, Branton D (2016). Three decades of nanopore sequencing. Nat Biotechnol.

[21] Wick R, Judd LM, Holt KE. Comparison of Oxford Nanopore basecalling tools; 2018.

[22] Li H (2018). Minimap2:pairwise alignment for nucleotide sequences. Bioinformatics.

[23] Simpson JT, Workman RE, Zuzarte PC, David M, Dursi LJ, Timp W (2017). Detecting DNA cytosine methylation using nanopore sequencing. Nat Methods.

[24] Liu H, Liu X, Zhang S, Lv J, Li S, Shang S (2016). Systematic identification and annotation of human methylation marks based on bisulfite sequencing methylomes reveals distinct roles of cell type-specific hypomethylation in the regulation of cell identity genes. Nucleic Acids Res.

[25] Yu G, Wang LG, He QY (2015). ChIPseeker: an R/Bioconductor package for ChIP peak annotation, comparison and visualization. Bioinformatics.

[26] Love MI, Huber W, Anders S (2014). Moderated estimation of fold change and dispersion for RNA-seq data with DESeq2. Genome Biol.

[27] Benjamini Y, Hochberg Y (1995). Controlling the false discovery rate: a practical and powerful approach to multiple testing. J Roy Stat Soc B.

[28] Benjamini Y, Yekutieli D (2001). The control of the false discovery rate in multiple testing under dependency. Ann Stat.

[29] Buenrostro JD, Giresi PG, Zaba LC, Chang HY, Greenleaf WJ (2013). Transposition of native chromatin for fast and sensitive epigenomic profiling of open chromatin, DNA-binding proteins and nucleosome position. Nat Methods.

[30] Buenrostro JD, Wu B, Chang HY, Greenleaf WJ (2015). ATAC-seq: A Method for Assaying Chromatin Accessibility Genome-Wide. Curr Protoc Mol Biol.

[31] Langmead B, Salzberg SL (2012). Fast gapped-read alignment with Bowtie 2. Nat Methods.

[32] Ramírez F, Ryan DP, Grüning B, Bhardwaj V, Kilpert F, Richter AS (2016). deepTools2: a next generation web server for deep-sequencing data analysis. Nucleic Acids Res.

[33] Zhang Y, Liu T, Meyer CA, Eeckhoute J, Johnson DS, Bernstein BE (2008). Model-based analysis of ChIP-Seq (MACS). Genome Biol.

[34] Ross-Innes CS, Stark R, Teschendorff AE, Holmes KA, Ali HR, Dunning MJ (2012). Differential oestrogen receptor binding is associated with clinical outcome in breast cancer. Nature.

[35] Hu QM, Tian HF, Xiao HB (2019). Effects of temperature and sex steroids on sex ratio, growth, and growth-related gene expression in the Chinese giant salamander *Andrias davidianus*. Aquat Biol.

[36] Hu QM, Meng Y, Tian HF, Zhang Y, Xiao HB (2016). Sexually dimorphic expression of foxl2 and ftz-F1 in Chinese giant salamander *Andrias davidianus*. J Exp Zool B Mol Dev Evol.

[37] Hu QM, Meng Y, Tian HF, Chen SL, Xiao HB (2015). Cloning, expression of, and evidence of positive election for, the prolactin receptor gene in Chinese giant salamander (*Andrias davidianus*). J Exp Zool B Mol Dev Evol.

[38] Hu QM, Xiao HB, Tian HF, Meng Y (2016). Characterization and expression of cyp19a gene in the Chinese giant salamander *Andrias davidianus*. Comp Biochem Physiol B Biochem Mol Biol.

[39] National Genomics Data Center and Partners (2021). Database Resources of the National Genomics Data Center, China National Center for Bioinformation in. Nucleic Acids Res.

[40] Tian HF, Hu QM, Li Z (2021). A high-quality de novo genome assembly of one swamp eel (*Monopterus albus*) strain with PacBio and Hi-C sequencing data. Gene.

[41] Chen S, Zhang G, Shao C, Huang Q, Liu G, Zhang P (2014). Whole-genome sequence of a flatfish provides insights into ZW sex chromosome evolution and adaptation to a benthic lifestyle. Nat Genet.

[42] Garcia-Moreno SA, Futtner CR, Salamone IM, Gonen N, Lovell-Badge R, Maatouk DM (2019). Gonadal supporting cells acquire sex-specific chromatin landscapes during mammalian sex determination. Dev Biol.

[43] Cheng H, Guo Y, Yu Q, Zhou R (2003). The rice feld eel as a model system for vertebrate sexual development. Cytogenet Genome Res.

[44] Lee HJ, Hou Y, Chen Y, Dailey ZZ, Riddihough A, Jang HS (2020). Regenerating zebrafish fin epigenome is characterized by stable lineage-specific DNA methylation and dynamic chromatin accessibility. Genome Biol.

[45] Lin CS, Park T, Chen ZP, Leavitt J (1993). Human plastin genes Comparative gene structure, chromosome location, and differential expression in normal and neoplastic cells. J Biol Chem.

[46] Wabnitz G, Balta E, Samstag Y (2017). L-plastin regulates the stability of the immune synapse of naive and effector T-cells. Adv Biol Regul.

[47] Qin H, Li X, Wang J, Sun G, Mu X, Ji R (2021). Ovarian transcriptome profile from pre-laying period to broody period of Xupu goose. Poult Sci.

[48] Sha S, Bhatia H, Yoon S (2018). An RNA-seq based transcriptomic investigation into the productivity and growth variants with Chinese hamster ovary cells. J Biotechnol.

[49] Zheng JP, Rudra-Ganguly N, Miller GJ, Moffatt KA, Roy-Burman P (1997). Steroid hormone induction and expression patterns of l-plastin in normal and carcinomatous prostate tissues. Am J Pathol.

[50] Shinomiya H (2012). Plastin family of actin-bundling proteins: its functions in leukocytes, neurons, intestines, and cancer. Int J Cell Biol.

[51] Hurd AJ, Whitmarsh GS, Baldwin SM, Kelly JP, Waltho NC, Price BA (1999). Hornby mechanism-based inhibition of C5-cytosine DNA methyltransferases by 2-H pyrimidinone. J Mol Biol.

[52] Mustapha UF, Assan D, Huang YQ, Li GL, Jiang DN (2022). High polymorphism in the dmrt2a gene is incompletely sex-linked in spotted scat, *Scatophagus argus*. Animals.

[53] Smith CA, Roeszler KN, Ohnesorg T, Cummins DM, Farlie PG, Doran TJ (2009). The avian Z-linked gene DMRT1 is required for male sex determination in the chicken. Nature.

[54] Boulanger L, Pannetier M, Gall L, Allais-Bonnet A, Elzaiat M, Le Bourhis D (2014). FOXL2 is a female sex-determining gene in the goat. Curr Biol.

[55] Jones P, Takai D (2001). The role of DNA methylation in mammalian epigenetics. Science.

[56] Zilberman D, Gehring M, Tran RK, Ballinger T, Henikoff S (2007). Genome-wide analysis of Arabidopsis thaliana DNA methylation uncovers an interdependence between methylation and transcription. Nat Genet.

