# Supplementary material for: Integrated chromatin accessibility and DNA methylation analysis to reveal the critical epigenetic modification and regulatory mechanism in gonadal differentiation of the sequentially hermaphroditic fish, Monopterus albus
Source: Biol Sex Differ. 2022 Dec 20;13:73. doi: 10.1186/s13293-022-00484-6 (PMC9764712; doi:10.1186/s13293-022-00484-6)
Supplement: Supplementary file 1 — Additional file 1: Fig. S1. Verification of the candidate genes. Fig. S2. In situ hybridization using sense probe. Table S1. Summary of the clean reads of the DNA methylation. Table S2. Summary of the ATAC-seq. Table S3. Distribution of peak of ATAC. Table S4. Differentially peak of ATAC in different group. Table S5. Primers and sequences used in this study. Table S6. Methylation level of each site for the ovary (OV), Ovotestis (OVT) and testis (TE) groups. Table S7. Methylation level of each site for the control and MT groups. Table S8. Methylation level of each site for the control and zebularine (ZE) groups. [file 13293_2022_484_MOESM1_ESM.doc]

**Integrated chromatin accessibility and DNA methylation analysis to reveal the critical epigenetic modification and regulatory mechanism in gonadal differentiation of the sequentially hermaphroditic fish, *Monopterus albus***

Qiaomu Hu*, Zitong Lian, Xueping Xia, Haifeng Tian, Zhong Li*

Yangtze River Fisheries Research Institute, Chinese Academy of Fishery Sciences, Wuhan 430223, China

Corresponding author:

Yangtze River Fisheries Research Institute Chinese Academy of Fishery Science Wudayuan First Road 8 430223 Wuhan China

Tel.: 0086-27-81780171; Fax: 0086-27-81780171

1. *mail address*: [hqmu0806@163.com (QM](mailto:hqmu0806@163.com (QM) H)

[lizhong@yfi.ac.cn (Z](mailto:lizhong@yfi.ac.cn(Z) L)

Table S1. Summary of the clean reads of the DNA methylation

|  | SumBase | SeqNum | N50Len | String Average Depth | Mapped | Ratio(%) |
| --- | --- | --- | --- | --- | --- | --- |
| Ovary | 36,111,583,077 | 3206602 | 18476 | 45.1 | 3195468 | 99.6528 |
| Ovotestis | 31,658,136,904 | 2140846 | 21515 | 39.5 | 2134133 | 99.5739 |
| Testis | 32,903,279,776 | 3797455 | 15808 | 41.1 | 3781275 | 99.5739 |

Table S2. Summary of the ATAC-seq

| **SampleID** | **Total_Reads** | **Mapped_Reads** | **Peak Number** |
| --- | --- | --- | --- |
| OVO36 | 110,192,628 | 107,129,886(97.22%) | 208,155 |
| OVO46 | 94,640,694 | 91,621,980(96.81%) | 183,899 |
| OVO48 | 110,784,604 | 107,387,630(96.93%) | 211,477 |
| OV3 | 120,839,559 | 117,701,591(97.40%) | 153,317 |
| OV4 | 110,211,467 | 107,320,018(97.38%) | 167,863 |
| OV6 | 106,563,036 | 104,086,402(97.68%) | 174,881 |
| TE5 | 106,161,935 | 103,627,863(97.61%) | 210,069 |
| TE6 | 107,210,678 | 104,630,398(97.59%) | 224,060 |
| TE7 | 118,789,241 | 115,094,959(96.89%) | 306,358 |

Table S3. Distribution of peak of ATAC.

| **Sample**  **ID** | Promoter  (%) | 3' UTR(%) | 5' UTR(%) | Exon(%) | Intron(%) | Distal Intergenic(%) |
| --- | --- | --- | --- | --- | --- | --- |
| OVO36 | 18.36 | 0 | 0 | 18.44 | 26.54 | 36.68 |
| OVO46 | 22.12 | 0 | 0 | 16.68 | 26.04 | 35.16 |
| OVO48 | 21.03 | 0 | 0 | 16.76 | 27.24 | 34.98 |
| OV3 | 35.74 | 0 | 0 | 12.58 | 28.39 | 23.29 |
| OV4 | 31.57 | 0 | 0 | 13.63 | 28.43 | 26.38 |
| OV6 | 28.34 | 0 | 0 | 11.82 | 29.07 | 30.77 |
| TE5 | 23.77 | 0 | 0 | 16.49 | 25.94 | 33.81 |
| TE6 | 21.58 | 0 | 0 | 16.66 | 26.86 | 34.89 |
| TE7 | 20.31 | 0 | 0 | 18.01 | 27.33 | 34.34 |

Table S4. Differentially peak of ATAC in different group.

| **Group** | **Total DARs** | **Increased DARs** | **Decreased DARs** |
| --- | --- | --- | --- |
| OVO/OV | 53,540 | 29,633 | 23,907 |
| TE/OV | 41,010 | 23,591 | 17,419 |

Table S5. primers and sequences used in this study

| Primers | Primer sequences(5’-3’) | Utilizations |
| --- | --- | --- |
| Mdnmt1S | GAACTCACTCGTGGTCTCCTATCTC | qRT-PCR |
| Mdnmt1A | CGAGTCTGGGCAACACCG | qRT-PCR |
| Mdnmt2S | CGACAGAAACCTTCAGCATCC | qRT-PCR |
| Mdnmt2A | TGGACTTCCGCTGTTGTTTCTA | qRT-PCR |
| plastin-2S | GATTGATGTGGACAACAACGGA | qRT-PCR |
| plastin-2A | GATGGTCGCTGGTTTTGGTC | qRT-PCR |
| EF-1a-F2 | CGCTGCTGTTTCCTTCGTCC | qRT-PCR |
| EF-1a-R2 | TTGCGTTCAATCTTCCATCCC | qRT-PCR |
| plastin-2 promethyS | GATTATAAATTTTTGTATGTATGGATG | DNA methylation |
| plastin-2 promethyA | TCGATAATACGTATTAAACTTTCCA | DNA methylation |
| plastin-2A-T7 | GATCACTAATACGACTCACTATAGCCTCCAGGATGTTCAGGGTGTAT | In situ hybridization |
| plastin-2S | GCTTTCGTTGCCAATCTGTTTA | In situ hybridization |
| plastin-2A | CCTCCAGGATGTTCAGGGTGTAT | In situ hybridization |
| plastin-2S-T7 | GATCACTAATACGACTCACTATAGGCTTTCGTTGCCAATCTGTTTA | In situ hybridization |
| plastin-2-3MUTdmrt1R | TATTCCATGTAGTTCTGACTCACATTACAGTTA | Site mutation |
| plastin-2-3MUTdmrt1F | TAACTGTAATGTGAGTCAGAACTACATGGAATA | Site mutation |
| plastin-2-3MUTfoxl2aR | TTTTGATATATCTAGTCAGTATGGTTCAAACTAATATATAACATT | Site mutation |
| plastin-2-3MUTfoxl2aF | AATGTTATATATTAGTTTGAACCATACTGACTAGATATATCAAAA | Site mutation |
| plastin-2-3MUTfoxl2bR | ACCACAGCGCCAGCTATCTACACGTAACTGATATATC | Site mutation |
| plastin-2-3MUTfoxl2bF | GATATATCAGTTACGTGTAGATAGCTGGCGCTGTGGT | Site mutation |
| pcDNA3.1-foxl2S | GGCGCTAGCATGATGGCATCTTACCAAAGCTT | Plasmid construction |
| pcDNA3.1-foxl2A | GGCGAATTCCTAAATATCAATCCTCGTGTGTAACG | Plasmid construction |
| pcDNA3.1-dmrt1S | GGCGCTAGCATGAACAAGGACAAGCAGCG | Plasmid construction |
| pcDNA3.1-dmrt1A | GGCGAATTCTTACTTGGTGGTGTCACCGTC | Plasmid construction |
| PGL3-Mn1S | GGCGGTACCGGTACCTCTGTCAGCTGACCC | Plasmid construction |
| PGL3-Mn1A | CGGCTCGAGCTCGAGCCTAACGACCTTTTG | Plasmid construction |
| PGL3-Mn2S | GGCGGTACCATTGGTGCGTTTTACCTTTTTACTT | Plasmid construction |
| PGL3-Mn2A | CGGCTCGAGCCTAACGACCTTTTGGACAGAGTAG | Plasmid construction |
| PGL3-Mn3S | GGCGGTACCAAAAATTTAATCAAAGGTAACAGAAATAC | Plasmid construction |
| PGL3-Mn3A | CGGCTCGAGCCTAACGACCTTTTGGACAGAGT | Plasmid construction |

Table S6. Methylation level of each site for the ovary(OV), Ovotestis(OVT) and testis (TE) groups

| Methylation site | OV | OVT | TE |
| --- | --- | --- | --- |
| 1 | 95.2 | 95.45 | 95.45 |
| 2 | 85.7 | 95.45 | 95.45 |
| 3 | 95.2 | 95.45 | 95.45 |
| 4 | 95.2 | 95.45 | 95.45 |
| 5 | 85.7 | 77.3 | 95.45 |
| 6 | 100 | 95.45 | 95.45 |
| 7 | 100 | 90.9 | 95.45 |
| 8 | 95.2 | 95.45 | 95.45 |
| 9 | 95.2 | 77.3 | 95.45 |
| 10 | 85.7 | 63.6 | 90.9 |
| 11 | 76.2 | 72.7 | 77.3 |
| 12 | 90.5 | 77.3 | 95.45 |
| 13 | 100 | 77.3 | 90.9 |
| 14 | 95.2 | 95.45 | 95.45 |
| 15 | 95.2 | 77.3 | 95.45 |
| 16 | 95.2 | 100 | 95.45 |
| 17 | 90.5 | 90.9 | 95.45 |
| 18 | 90.5 | 77.3 | 90.9 |
| 19 | 90.5 | 90.9 | 95.45 |
| 20 | 100 | 100 | 100 |
| 21 | 100 | 100 | 100 |

TableS7. Methylation level of each site for the control and MT groups

| Methylation site | C | MT |
| --- | --- | --- |
| 1 | 100 | 80 |
| 2 | 100 | 80 |
| 3 | 100 | 80 |
| 4 | 100 | 80 |
| 5 | 100 | 70 |
| 6 | 100 | 80 |
| 7 | 100 | 90 |
| 8 | 100 | 80 |
| 9 | 100 | 80 |
| 10 | 80 | 70 |
| 11 | 100 | 80 |
| 12 | 100 | 80 |
| 13 | 100 | 80 |
| 14 | 100 | 80 |
| 15 | 100 | 80 |
| 16 | 90 | 80 |
| 17 | 100 | 80 |
| 18 | 70 | 80 |
| 19 | 100 | 80 |
| 20 | 100 | 100 |
| 21 | 100 | 100 |

Table S8. Methylation level of each site for the control and ZE groups

| Methylation site | C | ZE |
| --- | --- | --- |
| 1 | 100 | 100 |
| 2 | 100 | 100 |
| 3 | 100 | 100 |
| 4 | 100 | 100 |
| 5 | 80 | 0 |
| 6 | 90 | 0 |
| 7 | 100 | 100 |
| 8 | 100 | 100 |
| 9 | 100 | 100 |
| 10 | 100 | 100 |
| 11 | 100 | 100 |
| 12 | 100 | 100 |
| 13 | 100 | 100 |
| 14 | 100 | 100 |
| 15 | 100 | 100 |
| 16 | 70 | 80 |
| 17 | 100 | 90 |
| 18 | 100 | 100 |
| 19 | 70 | 80 |
| 20 | 100 | 100 |
| 21 | 100 | 100 |


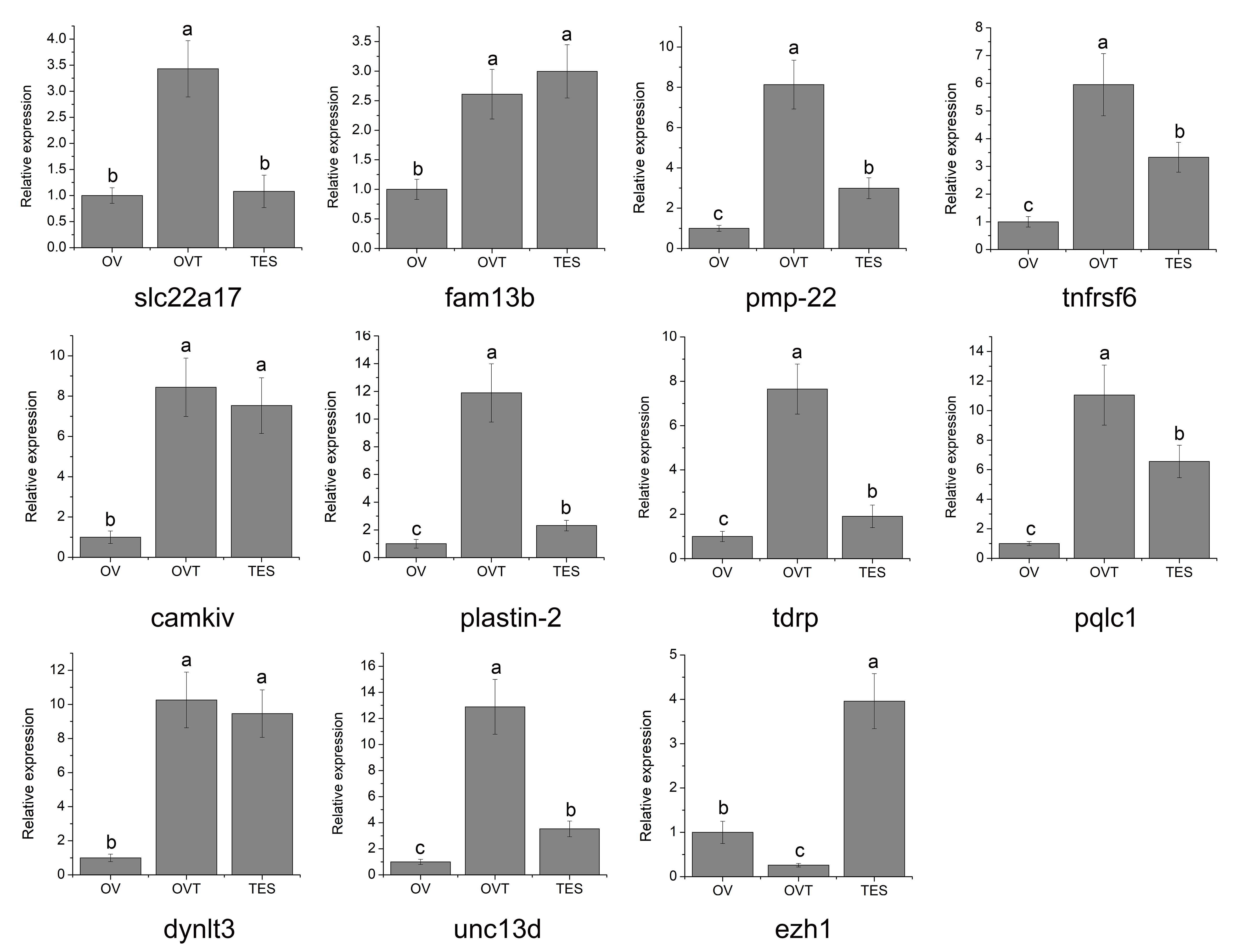


Fig S1


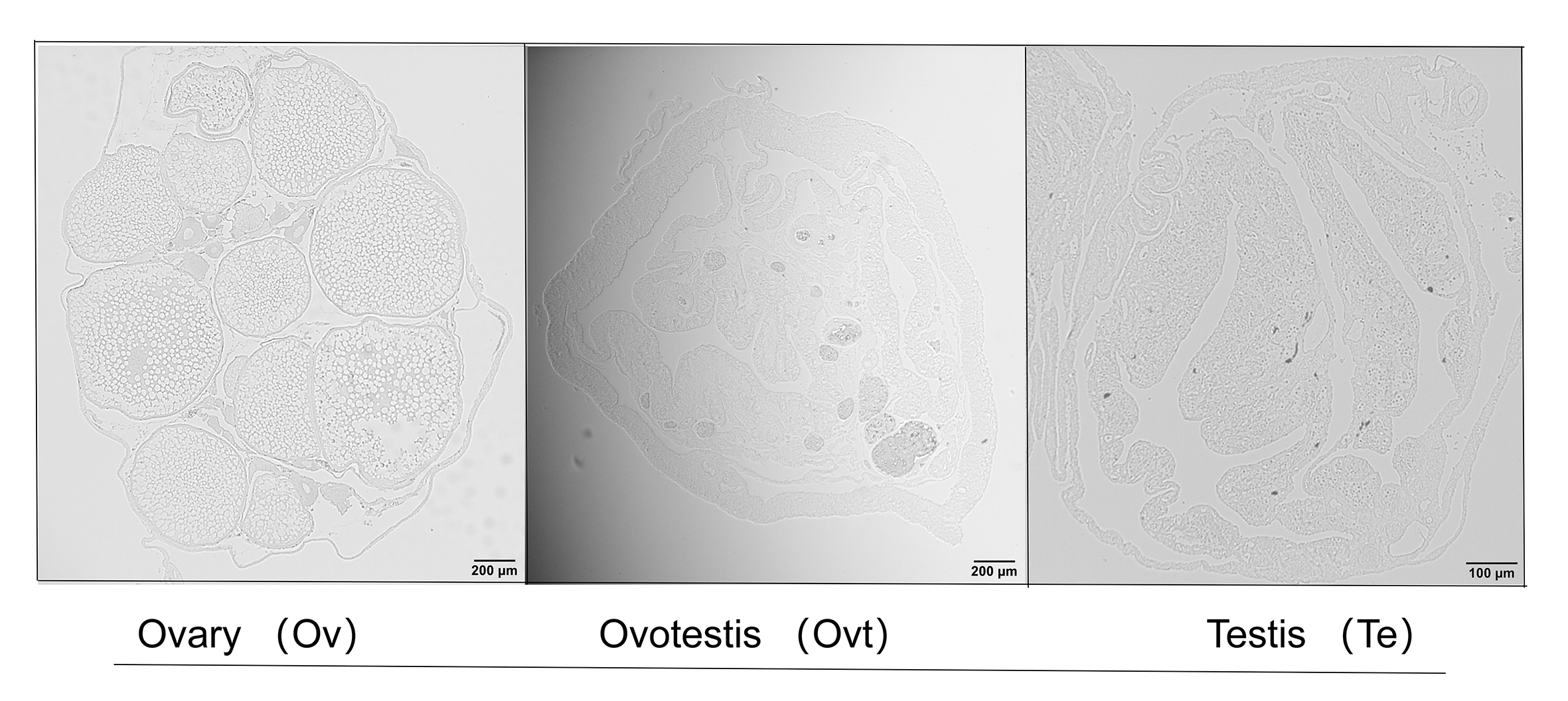


Fig S2
